# Effect of hydrokinesitherapy on balance and walking ability in post-stroke patients: A systematic review protocol

**DOI:** 10.1097/MD.0000000000013763

**Published:** 2018-12-21

**Authors:** Xin Wang, Taipin Guo, Tao Wang, Bo Jiang, Yan Su, Xiaoxia Tang, Jianglong Liao, Guanli Xie

**Affiliations:** a The Geriatitcs Hospital of Yunnan Province; b College of Rehabilitation Medicine, Yunnan University of Traditional Chinese Medicine; c Kunming Municipal Hospital of Traditional Chinese Medicine; d Kunming Municipal Hospital of Traditional Chinese Medicine, and The Jiang Bo Famous Medical Studio; e Kunming Municipal Hospital of Traditional Chinese Medicine, and Kunming Combination of Chinese and Western Medicine Minimally Invasive Spine Technology Center; f College of Rehabilitation Medicine, Yunnan University of Traditional Chinese Medicine, Kunming, Yunnan, China.

**Keywords:** balance, hydrokinesitherapy, protocol, stroke, systematic review, walking ability

## Abstract

**Introduction:**

Brain stroke is the second most common cause of death and major cause of disability in adults, representing a huge burden on patients and their families. Hydrokinesitherapy, a type of physical rehabilitation, may be beneficial to post-stroke recovery. We will systematically assess the clinical effectiveness and safety of hydrokinesitherapy for rehabilitation of stroke survivors in this review.

**Methods:**

We will perform a systematic search to identify all potentially relevant published studies on this topic. Online electronic databases including MEDLINE (via PubMed), EMBASE (via embase.com), Cochrane Central Register of Controlled Trials (CENTRAL) in the Cochrane Library, CINAHL (via EBSCOhost) and SPORTDiscus (via EBSCOhost) will be searched without language restrictions from their inception to September 30, 2018. All relevant randomized controlled trails (RCTs) will be screened according to predetermined inclusion criteria. Two independent reviewers will evaluate the methodological quality of each study included. One reviewer will extract data and another reviewer will check the accuracy. Any disagreements will be discussed with a third reviewer. The posture balance and walking ability will be defined as primary outcomes. Activities of daily living (ADL), drop-out and adverse events will also be assessed as secondary outcomes. The evaluation of methodological quality, data analysis will be completed using Cochrane Review Manager 5.3 according to Cochrane Handbook for Systematic Reviews of Interventions.

**Trial registration number:**

CRD42018110787.

## 1 Introduction

### 1.1 Description of condition

Stroke, a disruption of blood flow to the brain, is the second leading cause of death after ischemic heart disease worldwide estimated according to the Global Burden of Disease (GBD) 2010 Study.^[1]^ Although the ratio of stroke mortality and its mortality-to-incidence ratio has declined in the past two decades, the absolute number of affected people and survivors, related deaths, and disability-adjusted life-years (DALYs) lost are great and increasing.^[2]^ It is estimated that there will be almost 70 million stroke survivors, and more than 200 million DALYs lost globally by 2030.^[1]^ Stroke is also a primary cause of chronic impaired function, and postural balance disorders are the primary impairment after stroke.^[3]^ Such balance impairment may result in shorten supporting time and differences between two sides of the body, limited physical activity, and slowed walking speed, all of which reduce quality of life after stroke. Stroke requires an effective rehabilitation method for balance impairment after occurrence.

### 1.2 Description of the intervention

Hydrokinesitherapy, also called hydrotherapy, is a water-based exercise or aquatic exercise therapy. Interest in hydrokinesitherapy began some decades ago when it was used as a rehabilitation therapy for poliomyelitis.^[4]^ The success of this treatment indicated it could be used for patients with “weak muscles”. Hydrokinesitherapy has since been demonstrated to be beneficial for fitness and strength maintenance in various chronic conditions, including spinal cord injury,^[4]^ multiple sclerosis,^[5,6]^ cerebral palsy,^[7]^ arthritis,^[8]^ and Parkinson's disease (PD).^[9]^

Hydrokinesitherapy has also been used in stroke rehabilitation programme where it has been observed to be safe and suitable for stroke survivors.^[10]^ Previous studies have also suggested that hydrokinesitherapy programme promotes fitness,^[11]^ muscle strength,^[12]^ posture balance control,^[13]^ and reduces anxiety and depression scores^[14]^ in stroke survivors.

### 1.3 Possible mechanisms of hydrokinesitherapy

Hydrokinesitherapy may improve postural balance in stroke survivors due to its positive effects on lower extremity function, such as muscle force, pain and joint function, which are closely connected with equilibriums ability.^[15]^ Furthermore, the buoyant forces in water-based exercise result in a lowering of apparent body weight loaded on the lower extremity than on land.^[16]^ Additionally, the drag force exerted by water on the human body increases resistance to movement and allows an improvement of the motion of lower joints.^[15]^ As a consequence, buoyant force allows the stroke survivors to support their body easier in water than on land and perform movements more easily under the water, with diminishing the impulse forces loaded on the musculoskeletal system.^[15]^

Another possible benefit of the aquatic exercise therapy is easing of the reflex systems under water. More precisely, the sensitivity of the muscle spindle and skin was observed to be reduced during hydrotherapy.^[15]^ This led to a reduction in gamma fiber activity with a consequent reduction of muscle spasm and contractures, leading to a remission of pain and muscle relaxant effects.^[15]^

### 1.4 Importance of this review

The previous Cochrane systematic review on this topic published at 2011 indicated that there was not enough evidence to conclude whether the hydrokinesitherapy may reduce disability after stroke.^[10]^ In recent years, multiple clinical studies on the application of water-based exercise have been conducted and have demonstrated its safety and effects on the rehabilitation of stroke patients. Emerging data has also demonstrated that aquatic exercise might have the potential to improve posture balance,^[13,17–19]^ muscle strength,^[12,17,20]^ mobility,^[21]^ impaired cardiovascular fitness,^[11,22]^ activities of daily living (ADL) and mental impairments.^[14]^ Another study investigating the clinical effectiveness and safety of hydrokinesitherapy for rehabilitation of stroke survivors is ongoing.^[23]^ Therefore, it is necessary to carry out an updated systematic review to evaluate the efficacy and safety of water-based exercise for stroke survivors and provide additional clinical evidence for both patients and clinicians.

## 2 Methods

### 2.1 Design and registration

This protocol was registered in the international prospective register of systematic reviews (https://www.crd.york.ac.uk/PROSPERO/). Research registration unique identifying number is CRD42018110787. This protocol was performed in accordance with the Preferred Reporting Items for Systematic Reviews and Metaanalyses Protocols (PRISMA-P) statement.^[24]^ Formal ethical approval of this systematic review is not required as there is no direct human data involved.

### 2.2 Eligibility criteria

#### 2.2.1 Types of studies

We will only include clinical randomized controlled trials (RCTs) and we will exclude quasi-RCTs, non-RCTs such as case reports. For the randomized controlled cross-over trials, we will only analyze the first period as a parallel group trial. The language of publication will be limited to English without any restrictions on publication type.

#### 2.2.2 Types of participants

We will consider the trails involving patients who had suffered hemorrhagic or ischemic stroke regardless of age and gender. Stroke must be diagnosed according to the World Health Organization (WHO) definition,^[25]^ or a clinical definition of stroke when the WHO definition was not specifically stated, or confirmed by MRI or CT. The initial level of impairment will not be limited, regardless of the duration of stroke. If we find trials including participants with other type of injury (such as spinal cord injury or multiple sclerosis and cerebral stroke) we will only include RCTs with over 50% of patients suffering from stroke in our review.

#### 2.2.3 Types of interventions

Hydrokinesitherapy is broadly defined as any single or collective intervention involving participants being treated in the water. In order to differentiate between hydrokinesitherapy and using a spa pool or bathtub, we use the term “hydrokinesitherapy” to denote a physical activity that is structured, designed and reproducible. We will include only studies that used interventions implemented by a well-trained health care professional, such as a physical therapist. We will exclude the trials with interventions such as fumigation, soaking, spas, or mud baths.^[26]^ The duration and frequency of hydrokinesitherapy interventions will not be limited. However, stratification will be performed if there are sufficient studies to be included. Intervention in the experimental group should be hydrokinesitherapy (of any kind and irrespective of whether it is targeted to improve balance capacity and walking ability) for balance function, walking ability, muscle strength, ADL, and fitness of stroke survivors. The interventions in the control group will include the other non-water-based rehabilitation programme and no treatment. The studies comparing different types of hydrokinesitherapy will not be included in this review.

#### 2.2.4 Types of outcomes

##### 2.2.4.1 Primary outcomes

Patients’ balance function and walking ability will be defined as the primary outcomes in this review. Measurement scales that we will consider measures of equilibrium function include Berg Balance Scale (BBS),^[27]^ Time Up and Go Test (TUGT),^[28]^ Functional Reach Test (FRT),^[29]^ so on. Assessment tools that will be considered for walking ability are 10-meter walk test, 6-minute walk test, 2-minute walk test, walking speed, Rivermead Mobility Index, and so on.

##### 2.2.4.2 Secondary outcomes

The secondary outcome measures of this review will include ADL reported in the studies. Possible measure instruments for consideration include global assessment tools of ADL such as: Modified Barthel Index (MBI),^[30]^ Barthel Index (BI),^[31]^ Rivermead ADL Assessment,^[32]^ Modified Rankin Scale,^[33]^ Functional Independence Measure (FIM),^[34]^ and so on. Other secondary outcomes include drop out from the study during the treatment phase, and adverse events (including death from all causes).

#### 2.2.5 Search strategy

##### 2.2.5.1 Electronic searches

Five online electronic databases including Medline (PubMed), Embase (embase.com), Cochrane Central Register of Controlled Trials (CENTRAL), CINAHL (EBSCOhost), and SPORTDiscus (EBSCOhost) will be searched without language restrictions from their inception to September 30, 2018.

Search strategy for Medline via PubMed we have constructed is shown in Table 1 according to Cochrane Handbook for Systematic Reviews of Interventions.^[35]^ It will be adapted for other online electronic databases.

**Table 1:**
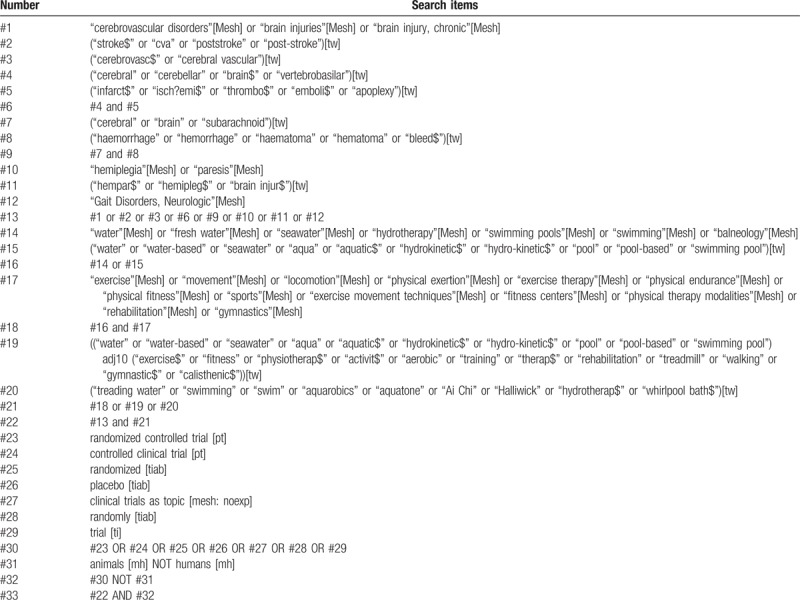
Search strategy for Medline via PubMed.

#### 2.2.6 Other resources

For the non-English language reports, we will also screen the abstracts if it is available in English.

#### 2.2.7 Data collection and analysis

##### 2.2.7.1 Selection of studies

**Figure 1:**
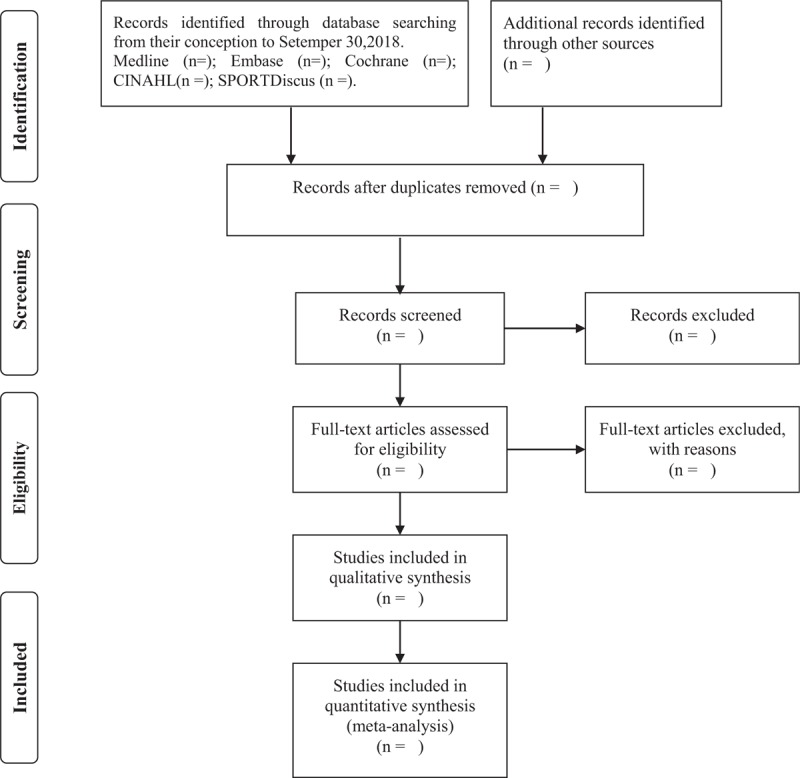
Flow diagram of the study selection process.

Reference management software, EndNote X7, will be used to manage all records. After removing duplicate records from the same report, 2 independent reviewers will screen the titles and abstracts of records to identify potentially eligible studies and eliminate obviously irrelevant reports. Two review authors will then read the full texts and include studies according to predetermined inclusion criteria. The studies that fitted the participants, intervention, study design (PIS) strategy of our review question will be included. If further information is needed to make a judgment, we will contact trial authors. We will exclude the trials that do not conform to our inclusion criteria. Any disagreements will be settled by consultation and discussion with a third reviewer. The PRISMA flow diagram (Fig. 1) will be used to report the details of the study selection process.

### 2.3 Data extraction and management

The information and outcome data from the selected trials will be extracted by one reviewer via a purpose-designed form and checked by another reviewer. The information extracted will contain base information about the publications (title of article, authors, published year, title of periodical, etc.), design (randomization, allocation concealment and blinding, etc.), patient characteristics (age, gender, inclusion/exclusion criteria, etc), interventions (experimental and control intervention, duration, frequency, intensity), and outcomes (measurement instruments, follow-up, drop-out and adverse events, etc). If missing data is observed, we will contact the original author. For continuous data, standard deviation (SD) or standard error (SE) values will be extracted. For categorical data, we will extract the number of events. The number of participants included in each analysis will also be extracted. For studies that include more than two arms, we will only extract data from the two groups that best match our comparative design (hydrokinesitherapy vs other rehabilitation or no-treatment). If there are two or more experimental groups (e.g., different duration or frequency of the hydrotherapy), we will split data from control group into parts of equal. For studies that have results from more than one time-point (e.g., repeated measurements), we will only extract the data from baseline and the final time-point. In order to meet the requirements of statistical conversions (e.g., standard error to standard deviation), spreadsheet software (Microsoft Excel) will be used to manage extracted data before entry to RevMan. Any disagreement regarding data extraction will be discussed and consulted with a third experienced reviewer.

### 2.4 Assessment of risk of bias

The “Risk of bias” table, recommended by the Cochrane Handbook for Systematic Reviews of Interventions,^[35]^ will be used to assess the methodological quality of the included studies by two independent reviewers.^[35]^ Reviewer authors’ judgments involve answering a specific question for each item.^[35]^ The items for the risk of bias tool are: random sequence generation (selection bias), allocation concealment (selection bias), blinding of participants and personnel (performance bias), blinding of outcome assessment (detection bias), incomplete outcome data (attrition bias), selective reporting (reporting bias) and other bias. Each included study will be categorized as having a high risk of bias (an answer “Yes”), unclear risk of bias (what happened in the study is unknown or insufficient detail is reported) and low risk of bias (an answer “No”) for each entry. Because it is impossible to blind study participants and personnel during the water-based exercise, the blinding of participants and personnel will be considered to be high risk of bias. Any discrepancies will be resolved via discussing and consulting with a third experienced reviewer.

### 2.5 Measure of treatment effect

For all measurement data (e.g., primary outcome: BBS), we will report mean differences (MDs) or standard MDs with 95% CIs. For all dichotomous data (e.g., secondary outcome: “drop-out”), risk ratios (RRs) with 95% CIs will be calculated.

### 2.6 Dealing with missing data

For missing data, we will contact the authors of the study to get additional information. If we fail to receive the required information, only the available data will be analyzed. We will discuss the potential impact of missing data on our review in the discussion section.

### 2.7 Assessment of heterogeneity

We will use the chi-squared test with a *P* value of .10 to assess heterogeneity and the I^2^ statistic to quantify heterogeneity of included studies. I^2^ values over 75% will be considered to represent substantial inconsistency. If a low level of heterogeneity is observed among the included studies, the results will be synthesized in a meta-analysis. Otherwise, we will only perform a qualitative analysis.

### 2.8 Assessment of publication bias

Funnel plots will be generated if more than 10 studies are included. If funnel plot asymmetry is observed, possible interpretations and language bias will be explored.

### 2.9 Data synthesis

The fixed effects model or random effects model will be adapted according to the results of heterogeneity. Cochrane Review Manager (Revman 5.3) will be used to perform data synthesis.

### 2.10 Subgroup analysis

If there is a sufficient number of randomized trails included, we will consider carrying out subgroup analysis when the heterogeneity results among studies was high (I^2^ > 75%). We intend to stratify by the origins of heterogeneity including characteristics of measurement tools (ie, balance function by BBS, TUG, FRT).

### 2.11 Sensitivity analysis

In order to test the robustness of evidence, sensitivity analysis will be performed to evaluate the influence of studies with a low methodological quality on the results. We will determine whether to exclude studies with lower quality based on the strength of evidence, number of participants and its influence on the pooled effective size.

### 2.12 Grading the quality of evidence

The Grading of Recommendations Assessment, Development and Evaluation (GRADE) system will be used to evaluate the quality of evidence for outcomes which rate the quality as very low, low, moderate, or high levels.^[36]^

## 3 Discussion

The previous Cochrane systematic review aimed to assess the effectiveness of water-based exercise on ADL of stroke patients was published in 2011.^[10]^ However, that review found that there was not enough evidence to conclude whether hydrokinesitherapy could reduce disability after stroke.^[10]^ Multiple new RCTs have been reported since publication of that review, with water-based exercise applied for stoke rehabilitation.^[12–14,19,20,22,23]^ It is, therefore, necessary to carry out an updated systematic review to revaluate the safety and efficacy of aquatic exercise therapy for stroke survivors. We hope that the results of this review may provide additional clinical evidence for both patients and clinicians. Such a review will also be helpful to make decisions regarding future practice of aquatic exercise therapy for stroke rehabilitation and will benefit for patient rehabilitation. However, there are also some limitations to this systematic review that should be noted. Firstly, studies published in English will only be included, which results in a language bias because some relevant studies published in non-English languages (eg, Chinese, Spanish, or German) may be excluded. Moreover, the diversity of styles, duration, and frequency of hydrokinesitherapy maybe results in a significant heterogeneity among the included studies.

## Acknowledgments

We would like to acknowledge Jing Li and her team at Chinese Cochrane Centre for their design assistance.

## Author contributions

JLL and GLX conceived and designed the study; XW and TPG made the manuscript preparation and wrote the draft manuscript; TW and BJ developed the search strategy; YS and XXT made the manuscript preparation.

**Conceptualization:** Jianglong Liao, Guanli Xie.

**Funding acquisition:** Bo Jiang.

**Methodology:** Xin Wang, Taipin Guo, Jianglong Liao, Guanli Xie.

**Project administration:** Yan Su, Xiaoxia Tang.

**Resources:** Tao Wang.

**Writing – original draft:** Xin Wang, Taipin Guo.

**Writing – review & editing:** Tao Wang, Bo Jiang, Yan Su, Xiaoxia Tang, Jianglong Liao, Guanli Xie.
